# JAK inhibitors for the treatment of inflammatory bowel disease: results of an international survey of perceptions, attitudes, and clinical practice

**DOI:** 10.1097/MEG.0000000000002650

**Published:** 2023-09-17

**Authors:** Sailish Honap, Peter M. Irving, Mark A. Samaan

**Affiliations:** a Department of Gastroenterology, St George’s University Hospital; b School of Immunology and Microbial Sciences, King’s College London; c IBD Centre, Guy’s and St Thomas’ NHS Foundation Trust, London, UK

**Keywords:** Crohn’s disease, Janus kinase inhibitor, safety, ulcerative colitis

## Abstract

**Background:**

Janus kinase inhibitors (JAKi) are small molecule drugs with demonstrated efficacy in inflammatory bowel disease (IBD). However, widespread utilisation may be hindered by safety concerns.

**Aims:**

This is the first study assessing risk-benefit perceptions and clinical practices of those using JAKi for IBD.

**Methods:**

A prospective, cross-sectional study was conducted using a 23-item survey distributed to IBD healthcare providers worldwide.

**Results:**

Of 385 respondents from 48 countries, 72% were tertiary-centre based and 50% were gastroenterologists with ≥10 years experience. JAKi were commonly used outside market authorisation (31%), though many (17%) were unconfident discussing JAKi risk-benefit profile and 7% had never prescribed JAKi. If venous thromboembolism risks were present, 15% preferentially referred for surgery than initiate JAKi; 21% would do this even if the patient was already anticoagulated. For patients relapsing on dose reduction, 8% would switch treatment rather than dose escalate. Conversely, 45% felt that cardiovascular safety concerns from post-marketing studies were irrelevant to IBD. Despite the lack of detailed, long-term safety data, safety profiles of JAK1-selective drugs were perceived to be favourable to tofacitinib by most (62%).

**Conclusion:**

The study indicates that while clinical practice appears to be in keeping with international guidance, a significant minority remain deterred by safety concerns.

## Introduction

Janus kinase inhibitors (JAKi) are small molecules that attenuate an array of pro-inflammatory cytokines signalling through the JAK-STAT pathway and are licenced for several immune-mediated inflammatory diseases. For inflammatory bowel disease (IBD), JAKi present a novel mechanism of action in an arena of significant unmet need; failure or intolerance rates of licensed medical treatments remain high and are associated with unacceptable rates of inappropriate corticosteroid use, hospitalisation, and surgery. JAKi have several advantages over other approved targeted therapies including their potency and rapidity of onset, lack of immunogenicity, and oral administration [1]. For ulcerative colitis (UC), tofacitinib, a partially selective JAKi, was first approved in 2018 followed more recently by the JAK1 selective drugs, filgotinib and upadacitinib. For Crohn’s disease (CD), upadacitinib was granted a licence by the UK’s Medicines and Healthcare products Regulatory Agency in February 2023 and by the European Medicines Agency (EMA) and Food and Drug Administration (FDA) in April and May 2023, respectively.

However, JAKi have been associated with numerous adverse events, findings that have largely been derived from post-marketing surveillance studies in rheumatology [2]. These include serious infection, major adverse cardiovascular events (MACE), venous thromboembolism (VTE), and malignancy, leading to safety warnings issued from the EMA and FDA. Both agencies have recommended measures to minimise the risk of serious side effects for all JAKi used to treat chronic inflammatory disorders [3,4]. The more concerning risks of MACE and cancer, however, have not been seen in IBD populations.

Anecdotally, the adverse safety profile and EMA guidance has led to uncertainty and anxiety among some IBD healthcare professionals (HCPs) regarding their use in IBD. Furthermore, the risk-benefit balance of JAKi in specific clinical scenarios such as patients with existing VTE remains uncertain. This study aimed to obtain an international perspective of the benefit-risk profile of JAKi as perceived by HCPs, which requires careful equipoise between using this expanding line of efficacious agents and the risk of side effects.

## Methods

### Study design

This was a prospective, cross-sectional, international study, which was conducted using a 23-item electronic web-based survey. The survey was peer-reviewed and endorsed by the Young ECCO (Y-ECCO), Clinical Research Committees (ClinCom) of ECCO and the ECCO governing board. A hyperlink to the survey was disseminated to all ECCO members and delegates of the 18th Congress of ECCO, 2023, held in Copenhagen, Denmark, and was active from 20 February 2023 until 31 March 2023. Methods of dissemination included newsletters via email to the whole ECCO membership, advertising through the ECCO Congress application, sharing on social media platforms, and viva voce among conference attendees. Completion of the anonymous voluntary survey was deemed to imply consent.

The survey was structured into four main sections: the first collected data on clinical practice characteristics of respondents, including role, country and setting of practice, and IBD workload. The second part focussed on the experience and confidence of using JAKi for IBD and identifying respondent perceptions of the benefit-risk profile. The third section evaluated current clinical practices to mitigate risk of/managing adverse events, and the final aspect assessed attitudes to the newer, more selective JAKi for the treatment of IBD. At the end of the study period, data were collated for analysis and interpretation. The complete survey is found in Supplementary Information 1, Supplemental digital content 1, http://links.lww.com/EJGH/A921.

### Study population, inclusion criteria, and definitions

Respondents were required to be HCPs actively managing IBD patients including doctors, specialist IBD nurses, and pharmacists. In the absence of a universal definition for an ‘IBD subspecialist’ and in accordance with previous studies, we pragmatically defined IBD subspecialists as gastroenterologists consulting ≥20 IBD patients weekly [5]. For sub-analyses, we defined junior gastroenterologists as having <10 years of practice, while senior gastroenterologists were defined as having ≥10 years of clinical experience. Other HCPs, which were likely to represent a minority of respondents, were not categorised according to experience.

### Statistical analysis

Descriptive statistics were performed where categorical variables were expressed as frequencies and percentages. When comparative analyses were performed, Likert responses of 1 and 2 (strongly disagree) and (disagree) were grouped together as responses refuting the presented clinical decision, while Likert responses of 4 (agree) and 5 (strongly agree) were grouped as supporting the proposed clinical decision. Responses of 3 (neither/undecided/lack of data) were analysed separately. A similar approach was used for questions assessing confidence among HCPs prescribing JAKi in various clinical scenarios. Thematic analyses were applied to the responses regarding typical patient profiles that JAKi were most and least likely to be used in.

Univariable analyses for group differences were performed using Fisher’s exact test for categorical data and the Mann–Whitney U test for continuous, non-parametric data. Significant variables on univariate analysis were then entered into multivariate analysis using binomial logistic regression. A *P*-value of < 0.05 was considered statistically significant. Statistical analyses were conducted using GraphPad Prism, version 9.5.1 for Mac, GraphPad Software, California.

## Results

### Study cohort

**Fig. 1.**
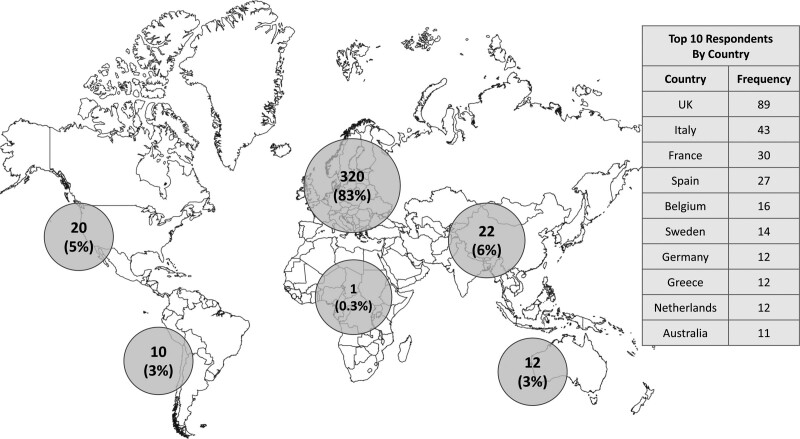
Geographical distribution of survey respondents, N (%).

In total, 850 participant responses were received, of which 385 were fully completed with no missing answers and included in the final analyses. There was representation from 48 countries across six continents. Most practiced in Europe, n = 320 (83%), followed by Asia, n = 22 (6%), and North America, n = 20 (5%) (Fig. 1). The majority were in-training or accredited gastroenterologists, n = 364 (95%), with some representation from IBD nurses, n = 10 (3%), and pharmacists, n = 9 (2%). A third of respondents, n = 139 (36%), were IBD ‘subspecialists’ according to our definition. This was a highly experienced cohort in the management of IBD that was predominantly based in tertiary IBD referral centres, n = 277 (72%), and consulted a median of 60 (IQR 30–100) unique IBD patients monthly. Half were senior gastroenterologists with ≥10 years’ experience, n = 192 (50%). Table 1 displays the characteristics of survey respondents.

**Table 1.** Characteristics of survey respondents

Characteristic	N = 385 (%)
Location of clinical practice	
Europe	320 (83)
North America	20 (5)
South America	10 (3)
Australasia	12 (3)
Asia	22 (6)
Africa	1 (0)
Clinical practice setting	
Public hospital (university affiliated)	240 (62)
Public hospital (general)	111 (29)
Private practice	34 (12)
Tertiary IBD referral centre	277 (72)
Type of healthcare provider	
GI physician	283 (74)
GI physician in training	81 (21)
GI surgeon	2 (1)
GI surgeon in training	0 (0)
IBD nurse specialist	10 (3)
IBD pharmacist	9 (2)
IBD subspecialist (>20 IBD cases/week)	139 (36)
Number of years practicing gastroenterology, y	
<5	112 (29)
6–9	81 (21)
10–19	135 (35)
>20	57 (15)
Total number of IBD patients seen monthly	
0–10	23 (6)
11–20	34 (9)
21–30	47 (12)
31–49	64 (17)
50–99	105 (27)
≥100	112 (29)

GI, gastrointestinal; IBD, inflammatory bowel disease.

### JAK inhibitors in clinical practice

#### Clinical setting and positioning

Thematic analyses were conducted of responses from survey participants for the typical patient profile in whom they would most and least likely prescribe a JAKi. Responses were grouped into patient, disease, and treatment characteristics, which identified that age, comorbidities, and disease refractoriness were the most common determinants of the decision to initiate a JAKi (Table 2). Typically, a JAKi was commenced if patients were younger, n = 257 (67%), with fewer comorbidities, n = 109 (29%), and biologic-experienced, n = 118 (31%). It is unclear whether the latter refers to restrictions from reimbursement policies or product licensing e.g., the US, or whether respondents reserve JAKi for medically refractory disease. Severe disease (for UC) was another common reason to commence a JAKi, n = 67 (17%). Conversely, older patients, n = 241 (63%), and those with comorbidities, n = 182 (47%), were least likely to be prescribed a JAKi. Where comorbidities were defined, MACE and VTE risk factors were most commonly reported but cancer risk factors and smoking status were both recorded in less than 10% of responses.

**Table 2.** Typical patient profiles of those most and least likely to be prescribed a JAK inhibitor as described by respondents

Most likely to prescribe	N = 385 (%)	Least likely to prescribe	N = 385 (%)
Patient characteristics		Patient characteristics	
Younger age	257 (67)	Older age	241 (63)
<65 specified	29 (8)	>65 specified	51 (13)
<60 specified	5 (1)	>60 specified	11 (3)
<55 specified	9 (2)	>55 specified	11 (3)
Fewer comorbidities	109 (28)	Comorbidities	182 (47)
MACE	52 (14)	MACE risk factors	100 (26)
VTE	39 (10)	VTE risk factors	90 (23)
Cancer	12 (3)	Cancer risk factors	35 (9)
Infection inc. shingles	2 (1)	Diabetes	9 (2)
EIMs	51 (13)	Obesity	13 (3)
Non-smoker	14 (4)	Smoking (current)	36 (9)
Male sex	38 (10)	Infection inc. shingles	10 (3)
No conception plans	10 (3)	Female sex	48 (12)
		Family planning specified	38 (10)
Disease characteristics		Hormonal treatment spec.	8 (2)
Severe disease (UC)	67 (17)	Biologic naive	11 (3)
ASUC	10 (3)		
Need for rapid induction	18 (5)	Drug characteristics	
Refractory disease	132 (34)	Adherence	4 (1)
Steroid-dependent	8 (2)		
Biologic-exposed	118 (31)		
TNF-exposed	94 (24)		
More than 2 biologics	35 (9)		
Drug characteristics			
Oral therapy	27 (7)		
Convenience specified	2 (1)		
Needle phobia specified	8 (2)		

JAKi were mainly positioned either second line, n = 136 (35%), or as a third line advanced therapy, n = 167 (43%). A significant minority, 27 (7%), of gastroenterologists had never prescribed JAKi despite consulting >100 unique patients annually and this was largely due to HCP-related safety concerns.

#### JAK inhibitor use outside of market authorisation

**Fig. 2.**
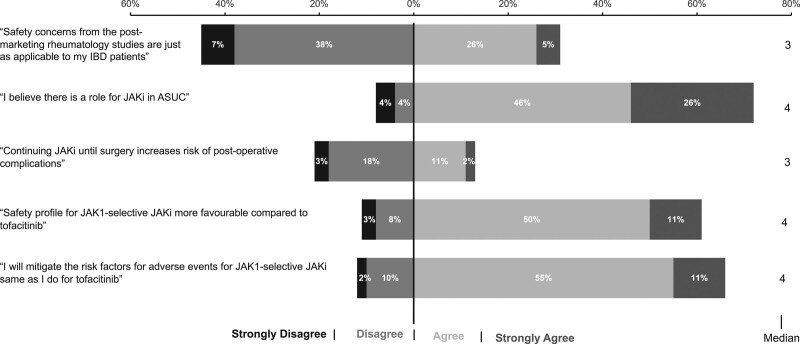
Survey responses assessing benefit-risk practices of JAK inhibitor use in inflammatory bowel disease.

Using JAKi off-label was common and just under a third of respondents, n = 121 (31%), prescribed a JAKi at least once for an off-label indication; this was detailed by 50 (41%) HCPs and included CD, n = 33 (66%), patients <18 years of age, n = 20 (40%), and acute severe UC (ASUC), n = 20 (40%). Most agreed (48%) or strongly agreed (26%) that there is a role for JAKi in the management of ASUC (Fig. 2). Use outside of product licence was more likely if the HCP was confident using JAKi, odds ratio (OR) 4.04 (95% confidence interval (CI) 1.97–9.03), *P* < 0.01, or if they regularly initiated JAKi (at least monthly), OR 1.91 (95% CI 1.15–3.18), *P* = 0.01. (Supplementary Information 2), Supplemental digital content 2, http://links.lww.com/EJGH/A922.

### Mitigating risk factors for adverse events

#### Dosing

In view of safety concerns, international recommendations advocate using the higher JAKi dose for the shortest duration possible, which is applicable to tofacitinib and upadacitinib but not filgotinib, which is licensed at a fixed dose through induction and maintenance. We explored prescribing practices in case of disease relapse for patients treated with a low-dose JAKi. Most respondents, n = 266 (66%), would dose escalate for a defined period and then attempt dose reduction. A quarter, n = 101 (26%), mostly consultants based at academic centres (80%), would use high-dose JAKi long-term without dose reduction. A minority, n = 30 (8%), would not attempt dose escalation to recapture response and instead would switch to an alternative advanced therapy.

#### Thrombosis, major adverse cardiovascular events, and non-melanoma skin cancer

We studied JAKi use in patients with existing VTE risk factors excluding active IBD. In line with guidance from the EMA, 224 (58%) respondents would avoid using a JAKi unless there were no other medical options available. Advantages of JAKi over other therapies mean that despite VTE risk factors, it may be clinically appropriate to position them ahead of other treatments and n = 102 (26%) reported they would do this. However, 59 (15%) HCPs would avoid a JAKi altogether and would instead refer for surgery. Equally, it may be appropriate to commence a JAKi for patients already established on anticoagulation, including ahead of other therapies, and 212 (55%) and 94 (24%) stated they would do this, respectively. In this scenario, however, a greater proportion, n = 79 (21%), would refer for surgery rather than use a JAKi. Multivariate analysis of respondents preferentially opting for a surgical approach highlighted that IBD subspecialists with full GI accreditation were less likely to do this, OR 0.40 (95% CI 0.17–0.85) *P* = 0.02, while those who do not routinely position JAKi first or second line were more likely to refer for surgery directly [OR 2.40 (95% CI 1.25–4.9), *P* = 0.01] (Supplementary Information 2, Supplemental digital content 2, http://links.lww.com/EJGH/A922).

Clinical practice of managing JAKi-related dyslipidaemia and how HCP perceive the increased MACE risk in their IBD patients is unclear, particularly given that IBD patients are younger and without traditional cardiovascular risk factors. In the event of post-induction dyslipidaemia, the vast majority of participants would reassess the cardiovascular risk profile and either commence or refer for lipid-lowering therapy, n = 274 (71%). Some would adopt a watch-and-wait approach and carefully monitor the lipid profile, n = 82 (21%). However, increased risk of MACE identified in a post-marketing study did not concern almost half of the cohort who either disagreed or strongly disagreed that these issues were relevant to their IBD patients, n = 174 (45%) (Fig. 2).

Regarding the link between JAKi and non-melanoma skin cancer (NMSC), the overwhelming majority were aware of this risk and recommended sun protection to their patients, n = 310 (81%). Of these, 248 (80%) additionally recommended periodic skin examination. Only a minority, n = 40 (10%), were unaware of this link. Those who had a high IBD caseload and consulted ≥20 IBD patients weekly were more likely to inform patients of the risk of NMSC [89% vs. 70% (*P* < 0.01)] and were also more likely to recommend regular skin checks [72% vs. 55% (*P* < 0.01)].

#### Herpes zoster infection

Clinical practice for mitigating the risk of varicella zoster reactivation with JAKi was varied. Most were able to ensure the inactivated vaccine was administered either prior to commencing JAKi, n = 159 (41%), or at some point during treatment, n = 54 (14%). However, a quarter of the cohort were unable to vaccinate their IBD patients due to restricted vaccine availability, n = 102 (26%). Although the vaccine type was not specified in the question, it is likely this refers to the inactivated (non-live) shingles vaccine, which is not widely available in some areas. No clear geographical trends with respect to vaccine availability were identified. Only a minority, n = 41 (11%), did not routinely attempt to vaccinate their patients as they felt the risk of shingles was too small to consider vaccination.

### Perception of benefit-risk profile of JAK inhibitors

#### Discussing JAKi benefit-risk profile with patients

**Fig. 3.**
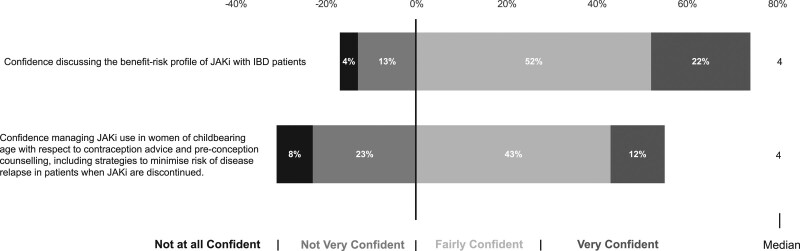
Survey responses assessing confidence in prescribing JAK inhibitors in inflammatory bowel disease.

Overall, 285 (74%) HCPs were either ‘very confident’ or ‘fairly confident’ with discussing the benefit-risk profile of JAKi use in IBD; 64 (17%) respondents, mainly accredited gastroenterologists, did not feel confident (Fig. 3). JAKi are contraindicated in pregnancy and during lactation due to teratogenicity seen in pre-clinical studies at high JAKi doses, although evidence in humans is sparse. Women of childbearing age may require counselling if JAKi are being considered. This is also applicable to patients already established on JAKi who wish to conceive in whom it is important to discuss strategies to recapture response in the event of disease relapse upon JAKi discontinuation. Again, most respondents were confident with having this discussion, n = 211 (55%), but a third were not, n = 120 (31%). IBD subspecialists as defined by IBD caseload and senior HCP with ≥10 years’ experience felt more confident having these discussions (*P* < 0.01 for all comparisons; Table 3).

**Table 3.** Perception and practices of healthcare professionals based on IBD patient caseload and seniority

	High IBD caseload^a^	Low IBD caseload	*P*-value	Senior HCP	Junior HCP	*P*-value
N	217	168		192	193	
Median years in practice (IQR)	11 (7–18)	7 (4–12)	**<0.01**	15 (12–20)	5 (3–7)	**<0.01**
Tertiary referral centre, n (%)	169 (78)	108 (64)	**<0.01**	133 (69)	144 (75)	0.26
University affiliated hospital, n (%)	147 (68)	93 (55)	**0.01**	113 (59)	127 (66)	0.17
IBD consults/month, median (IQR)	100 (60–170)	30 (20–40)	**<0.01**	64 (40–121)	40 (25–80)	**<0.01**
Prescribing JAKi at least monthly, n (%)	104 (48)	57 (34)	**0.01**	81 (42)	80 (41)	0.92
Confident in JAK prescribing, n (%)	183 (84)	102 (61)	**<0.01**	156 (81)	129 (67)	**<0.01**
Confident in JAK use in pregnancy, n (%)	137 (63)	74 (44)	**<0.01**	122 (64)	89 (46)	**<0.01**
Uses JAKi off-label, n (%)	75 (35)	46 (27)	0.15	58 (30)	63 (33)	0.66
Initiates JAKi alongside anticoagulation (if appropriate), n (%)	52 (24)	42 (25)	0.81	53 (28)	41 (21)	0.16
Initiates JAK in those with VTE risk factors (excluding active IBD) if appropriate, n (%)	61 (28)	41 (24)	0.48	52 (27)	50 (26)	0.82
Informs patients of JAKi-related NMSC risk, n (%)	193 (89)	117 (70)	**<0.01**	162 (84)	148 (77)	0.07
Informs patients of JAKi-related NMSC risk and advises routine skin checks, n (%)	156 (72)	92 (55)	**<0.01**	128 (67)	120 (62)	0.39
Uses long-term high-dose JAKi to maintain remission for relapse on dose reduction, n (%)	61 (28)	40 (24)	0.35	52 (27)	49 (25)	0.73

Bold values indicate statistical significance.

a High IBD caseload was defined as healthcare professionals who consulted ≥20 IBD patients weekly.

#### Post-operative complications following JAKi therapy

Responses to assess perception of JAKi and risk of post-operative complications were equivocal with two-thirds of the cohort providing a neutral response to indicate they were undecided due to a lack of data in this area. The proportion of respondents refuting and supporting the provided statement were evenly balanced (Fig. 2).

#### Perception of selective JAK inhibitors

The safety profiles of the JAK1 selective drugs, filgotinib and upadacitinib, were perceived to be more favourable than tofacitinib by most, n = 238 (62%) (Fig. 2). Despite this, a similar proportion of respondents remained cautious and would mitigate the risk factors for adverse events in the same way that they currently do for tofacitinib, n = 253 (66%).

## Discussion

To the best of our knowledge, this is the first study to examine perceptions and clinical practices of healthcare providers who prescribe JAKi in IBD. The study provides unique insights from an international cohort including highly experienced IBD clinicians. While JAKi provide a convenient oral option that has revolutionised management of several immune-mediated diseases, the class-wide safety concerns have significant implications on how they are perceived and used in clinical practice. Our results indicate that while clinical practice appears to be in keeping with international guidance, a significant minority are deterred by the safety profile.

Most adverse events related to JAKi are mild to moderate, predictable, and easy to manage. However, the increased incident rates (IR) of MACE and malignancy with tofacitinib and baricitinib have led to restrictions from the EMA and FDA [2]. These include reserving all JAKi as a last-line medical option in; those ≥65 years of age, current or significant ex-cigarette smokers, and those with risk factors for cardiovascular disease or malignancy [6]. We show that the typical patient profiles for those initiated on a JAKi centre on age and comorbidities and that JAKi are usually reserved for biologic-experienced patients. Interestingly, a history of cancer and smoking discouraged <10% of respondents highlighting limitations of knowledge and interpretation even among IBD specialist respondents.

Chronic systemic inflammation is a risk factor for MACE and is driven by accelerated atherosclerosis [7]. IBD patients have a modestly increased risk of cardiovascular events above the general population, with an increased risk of arterial thrombotic events during periods of active disease [8–10]. Therefore, patients should be counselled on aggressive risk factor modification. The increased IR of MACE has not been demonstrated with JAKi in the IBD population [11]. This may be due to a younger comorbidity-free population in IBD than those with rheumatoid arthritis and may explain why under half the survey respondents felt that the findings from the post-marketing surveillance studies were not applicable to patients they treat. Despite this, most would either commence a statin or adopt a careful monitoring approach in those that develop JAKi-induced dyslipidaemia. JAKi are widely associated with dose-dependent elevations of HDL and LDL cholesterol without affecting the HDL:LDL ratio, but these changes are reversible on treatment cessation or use of statins [12].

Patients with active IBD have a twofold increased risk of VTE [13]. However, a meta-analysis including 10 controlled studies and 5143 JAKi-exposed patients did not find significant differences in the risk of VTE with the use of JAKi in patients with immune-mediated inflammatory diseases (RR 0.90, 95% CI 0.32–2.54) [14]. The EMA have advised that JAKi should be used with caution in those with VTE risk factors and patients should therefore be screened for other concurrent VTE risk factors [15]. In this eventuality, we show that most HCP would opt for an alternative advanced therapy. A minority, likely deterred by the safety profile, would avoid a JAKi in the absence of other medical options and be referred for surgery, even if the patient was already anticoagulated. The fact that respondents would preferentially refer for surgery, which carries a significant risk of thrombosis and other complications, perhaps further highlights the guarded approach taken by a significant minority with this drug class.

Whether JAKi treatment increases the risk of peri- or post-operative complications is currently unclear, and this was supported by a substantial number of neutral responses in this study. Regardless, the short half-life of JAKi used in IBD ranges from 3 to 14 h and is a potential advantage in circumstances where abrupt cessation is required, such as in the pre-surgical setting or in the context of an infection.

Aggressive squamous cell carcinomas have been reported with JAKi, with older age, previous NMSC, and prior anti-TNF failure associated with increased NMSC risk [16,17]. The product labels for all JAKi advise periodic skin examination for all patients, particularly those at higher risk of skin cancer. However, the IR of NMSC across the clinical trial programmes for JAKi were low at 0.51/100 patient-years; IR among patients exposed to comparator (mainly placebo) was 0.27/100 patient-years [14]. Nonetheless, the overwhelming majority of survey respondents advise sun protection and regular skin examination to their JAKi treated patients.

JAKi use outside of marketing authorisation was pervasive with CD and ASUC being the commonest reasons for off-label use. Neither tofacitinib nor filgotinib are licensed for CD as they failed to meet their primary endpoints in phase 2 and 3 studies [18,19]. CD was classed as off-label indication despite upadacitinib obtaining a UK license just prior to this survey distribution; it is not expected to be widely available for CD until mid-2023. The European Commission approval on the other hand was announced after the survey closure [20]. The positive results from the upadacitinib phase III programme in early 2022 may have encouraged pre-licensing use in CD if local policies allowed this. Off-label JAKi use for the treatment of ASUC is unsurprising given the potency and rapidity of onset of JAKi together with the accumulating evidence for their use in this setting [21,22]. Outcomes from the clinical trials of tofacitinib in ASUC are eagerly awaited [23,24]. It is important to note that off-label JAKi use based on prescribed confidence is a subjective measure so these findings should be interpreted with a degree of caution.

This study showed that JAK1-selective inhibitors were perceived by most to be safer than tofacitinib although no conclusive evidence exists thus far to demonstrate that selectivity confers a more favourable safety profile. Ongoing analyses of long-term data from randomised and real-world cohorts are needed to better understand whether a meaningful clinical difference in safety and efficacy exists between pan-JAK and selective JAK inhibition. While pooled safety analyses so far are encouraging, the absence of detailed long-term safety data has led the EMA to apply restrictions across the drug class [25,26]. The clinical practice of respondents to this survey is in line with this as most HCP mitigate for risk factors for upadacitinib and filgotinib in the same way they do for tofacitinib.

The strength of this study includes its originality given the dearth of literature regarding clinical practices and perceptions of JAKi for the management of immune-mediated diseases. Secondly, there was a large sample size of respondents with global representation. Most were IBD subspecialty gastroenterologists practicing in tertiary referral centres and experienced in the use of JAKi providing insights into their practices. Limitations include an uneven continental distribution of survey responses; most responses were from European countries. Secondly, there was a selection bias as participants were derived from a single specialist congress and therefore not truly representative of, nor generalisable to, a wider denominator of lesser experienced clinicians and hospitals whose knowledge and influence may affect JAKi prescribing. Thirdly, 21% of responses came from fellows who may not have had final decision on prescribing. However, it is likely that these practices and perceptions may then be integrated into practice as a specialist. The final limitation of this study was the inability to derive a response rate for this survey, which may have introduced a degree of response bias. The final denominator was unknown given the inability to calculate the precise number of invitations based on the dissemination methods employed.

In conclusion, our study provides a careful assessment of the benefit-risk profile of JAKi as perceived by HCPs. While most are assured about their use in IBD, a minority remain discouraged with concerns regarding the safety profile. With ‘black box warnings’ and regulatory restrictions in the limelight since the introduction of JAKi in IBD, for some, the potential over-reaction to safety concerns with this class of drugs may take several years to rectify. Approaches to mitigate these risk factors are broadly similar and incorporate the most recent recommendations from the EMA. Although the potential clinical significance of selectively inhibiting different JAK isoforms remains to be unravelled, JAK1-selective drugs are perceived to be safer. Personalised stratification to appropriate JAKi use is an attractive future goal.

## Acknowledgements

We thank committee members of Y-ECCO, ClinCom, ECCO governing board, and the administrative team in the ECCO offices for their assistance with revising, endorsing, and then distributing the survey to the ECCO members and conference delegates. We also thank the healthcare professionals who participated and completed this survey.

SH and MAS conceived the idea for the study. SH designed the survey, analysed, and interpreted the results, and prepared the draft manuscript. All authors critically reviewed and revised the manuscript, and all authors approved the final manuscript as submitted and agree to be accountable for all aspects of the work.

Guarantor of the article: Dr Sailish Honap.

The authors confirm that the data supporting the findings of this study are available upon request.

### Conflicts of interest

SH served as a speaker, a consultant, and/or an advisory board member for Pfizer, Janssen, AbbVie, and Takeda. Travel grants received from Ferring, Falk Pharma, and Pharmacosmos. Research supported by Pfizer. PMI has received lecture fees from AbbVie, Warner Chilcott, Ferring, Falk Pharma, Takeda, MSD, Johnson and Johnson, Shire and Pfizer. Financial support for research: MSD, Takeda, and Pfizer. Advisory fees: AbbVie, Warner Chilcott, Takeda, MSD, Vifor Pharma, Pharmacosmos, Topivert, Genentech, Hospira, Samsung Bioepis. MAS served as a speaker, a consultant, and/or an advisory board member for Sandoz, Janssen, Takeda, MSD, Falk, AbbVie, Bristol Myers Squibb, Galapagos, Pfizer, and Samsung Bioepis.

## Supplementary Material
