# Supplementary material for: JAK inhibitors for the treatment of inflammatory bowel disease: results of an international survey of perceptions, attitudes, and clinical practice
Source: Eur J Gastroenterol Hepatol. 2023 Sep 17;35(11):1270–7. doi: 10.1097/MEG.0000000000002650 (PMC10659244; doi:10.1097/MEG.0000000000002650)
Supplement: Supplementary file 1 [file ejgh-35-1270-s001.pdf]

European Crohn's and Colitis Organisation

Perceptions, Attitudes, and Practices Regarding the Benefit-Risk Profile of Janus Kinase Inhibitors for the Treatment of Inflammatory Bowel Diseases

Introduction

Janus kinase inhibitors (JAKi) are small molecule drugs that attenuate an array of pro-inflammatory cytokines that signal through the JAK-STAT pathway, leading to numerous trials assessing efficacy across the spectrum of immune-mediated inflammatory diseases. The knowledge about their safety profile is mainly based on data from the rheumatological setting, however, it has led to uncertainty and anxiety among IBD healthcare professionals regarding their use in IBD.

JAKi present a novel mechanism of action in an arena of significant unmet need in IBD; failure or intolerance rates of licensed medical treatments remain high, with unacceptable rates of corticosteroid use, hospitalizations, and surgery. JAKi have several advantages over other approved targeted therapies including their potency and rapidity of onset, lack of immunogenicity, more predictable pharmacokinetics, and oral administration. For ulcerative colitis (UC), tofacitinib, a partially selective JAKi, was the first to be approved in 2018 followed more recently by the JAK1 selective drugs, filgotinib and upadacitinib; results of late-stage trial programs for Crohn's disease (CD) also appear promising.[1]

However, JAKi have been associated with numerous adverse events. These include serious infection, major adverse cardiovascular events, venous thromboembolism (VTE), and malignancy, leading to safety warnings issued from the European Medicines Agency (EMA) and Food and Drug Administration (FDA). Further, the EMA has recently recommended measures to minimize risk of serious side effects for all JAKi used to treat chronic inflammatory disorders.[2]

Aims

This survey aims to obtain an international perspective of the benefit-risk profile of JAKi perceived by healthcare providers, which requires careful equipoise between using this expanding line of efficacious agents and the risk of side effects. The survey will focus on:

- Identifying clinician perceptions on the benefit-risk profile and confidence in initiating JAKi
- Assessing current clinical practices to mitigate risk of/managing adverse events
- Assessing attitudes to newer JAKi for IBD

Methodology

We seek to liaise with the Y-ECCO and ClinCom committees to distribute this anonymous questionnaire to the delegates of the 18th Congress of ECCO 2023 in Copenhagen, Denmark. Categorical variables will be described as raw numbers, proportions or percentages and comparison of groups will be made using Chi-squared test. Statistical significance will be set at a p-value threshold of <0.05.

References

1. Upadacitinib (RINVOQ®) Achieved Clinical Remission and Endoscopic Response at One Year in Phase 3 Maintenance Study in Patients with Crohn's Disease. AbbVie News Center [Internet]. [Accessed November 2022]. Available from: https://news.abbvie.com/news/press-releases/upadacitinib-rinvoq-achieved-clinical-remission-and-endoscopic-response-at-one-year-in-phase-3-maintenance-study-in-patients-with-crohns-disease.htm
2. EMA recommends measures to minimise risk of serious side effects with Janus kinase inhibitors for chronic inflammatory disorders | EMA [Internet]. Accessed 4th November 2022. Available from: https://www.ema.europa.eu/en/medicines/human/referrals/janus-kinase-inhibitors-jaki

European Crohn's and Colitis Organisation

Perceptions, Attitudes, and Practices Regarding the Benefit-Risk Profile of Janus Kinase Inhibitors for the Treatment of Inflammatory Bowel Diseases

Please complete this anonymous survey on using JAKi in IBD

Clinical Practice and Setting

1. How many years have you been practicing gastroenterology? ____________________

2. Which country do you currently practice in? __________________________

3. In what setting is your practice primarily based?

   ❑ Public hospital (university affiliated) ❑ Public hospital (general) ❑ Private practice

4. Is your practice based at a tertiary referral center for the treatment of IBD?

   ❑ Yes ❑ No

5. Please indicate the best description of your role:

   ❑ GI physician in training ❑ GI physician ❑ GI surgeon in training ❑ GI surgeon
   ❑ IBD nurse specialist ❑ Other non-medical prescriber (please specify) _____________

6. Approximately how many unique UC and CD patients do you personally consult per month at your center?

   UC ____________ CD ____________

Using JAK Inhibitors in Clinical Practice

7. How frequently do you initiate patients on JAKi for the treatment of IBD? (select one)

   ❑ At least weekly ❑ At least monthly ❑ At least quarterly ❑ Six monthly or less ❑ None

8. In which setting have you used JAKi for the treatment of IBD? (tick one)

   ❑ Off-label* and licensed indication ❑ Licensed indication (UC) only
   ❑ Not used due to lack of availability ❑ Not used due to safety concerns

   *Please specify off-label use _________________________________

   *Off-label use includes, but not limited to, acute severe UC, CD, use in pediatrics, concomitant use of a monoclonal antibody

9. How confident are you discussing the benefit-risk profile of JAKi with IBD patients? (select one)

   ❑ Not at all confident ❑ Not very confident ❑ Neither ❑ Fairly confident ❑ Very confident

10. The increased incident rate of major adverse cardiovascular events and malignancy compared to anti-TNF drugs seen in a safety trial among rheumatology patients aged >55 and at least one cardiovascular risk factor is just as applicable to my IBD patient population? (select one)

❑ Strongly disagree ❑ Disagree ❑ Undecided ❑ Agree ❑ Strongly agree
❑ Unaware of safety outcomes from this trial

11. Regarding treatment positioning for advanced therapies (excluding 5-ASA, corticosteroids, immunomodulators), I usually use JAKi…(select one)

❑ First line ❑ Second line ❑ Third line ❑ Avoid unless no other medical alternative available

12. Please describe the patient profile in whom you would most and least likely use a JAKi (consider factors such as age, comorbidities (including EIMs), response to previous therapies etc)

Most likely to use JAKi ____________
Least likely to use JAKi ____________

13. In the event of disease relapse on JAKi dose reduction during maintenance, my treatment strategy is to (select one)

❑ Dose escalate and use the higher dose long term as maintenance
❑ Dose escalate and use the higher dose for a defined period, and re-attempt dose reduction
❑ Consider an alternative advanced therapy

14. I believe there is a role for the use of JAKi in the treatment of acute severe UC.

❑ Strongly disagree ❑ Disagree ❑ Undecided ❑ Agree ❑ Strongly agree

Mitigating Risk Factors for Adverse Events

15. How do you mitigate for the increased risk of herpes zoster infection (shingles) for *most* of your patients? (select one)

❑ Administer live vaccine prior to therapy ❑ Administer inactivated vaccine prior to therapy
❑ Administer inactivated vaccine during therapy ❑ Unable to vaccinate due to limited vaccine availability/restricted access for patients I treat ❑ I don't-risk too small to consider vaccination

16. In patients with existing risk factor(s)* for VTE (excluding active IBD), do you? (select one)

❑ Avoid JAKi altogether and refer for surgery
❑ Avoid JAKi unless no other medical alternative available
❑ Initiate JAKi with thromboprophylaxis for duration of VTE risk factor
❑ Initiate JAKi with careful counselling of VTE risk but without thromboprophylaxis

*VTE risk factors include, but not limited to, previous VTE, known thrombophilia, active cancer, recent major surgery, prolonged immobility, age >60, estrogen therapy, postpartum, obesity

17. In patients already taking anticoagulation to prevent/treat systemic or venous thromboembolism, do you? (select one)

❑ Avoid JAKi altogether and refer for surgery
❑ Avoid JAKi unless no other medical alternative available and initiate alongside anticoagulation
❑ Initiate JAKi alongside concomitant anticoagulation ahead of other advanced IBD therapies if appropriate

18. In those continuing JAKi until surgery, there is an increased risk of post-operative complications (e.g. infection, VTE) (select one)

❑ Strongly disagree ❑ Disagree ❑ Agree ❑ Strongly agree
❑ Undecided/Not enough data available

19. JAKi are associated with hyperlipidemia, including total cholesterol (TC) and non-HDL cholesterol. For patients with elevated post-induction lipid parameters compared to baseline, which of the following are you most likely to do in your clinical practice? (select one)

❑ Stop JAKi and switch class unless no other medical alternative available
❑ Reassess CV risk profile and if necessary, commence/refer for lipid lowering treatment
❑ Continue JAKi and adopt closer monitoring of lipid parameters
❑ Continue JAKi without further monitoring of lipid profile

20. Non-melanoma skin cancers (NMSC) have been reported in patients treated with JAKi. In my clinical practice, for patients at higher risk of NMSC, I usually... (select one)

❑ Inform patients of this risk, advise sun protection, and recommend periodic skin examinations
❑ Inform patients of this risk and advise sun protection
❑ Do not inform patients but I am aware of the association of NMSC and JAKi
❑ Do not inform patients as I was not aware of the association of NMSC and JAKi

21. JAKi are contraindicated in pregnancy and during lactation due to their teratogenic potential. I am confident in managing JAKi use in women of childbearing age with respect to contraception advice and pre-conception counselling for those already established on a JAKi, including strategies to minimize risk of disease relapse in patients when JAKi are discontinued.

❑ Not at all confident ❑ Not very confident ❑ Neither ❑ Fairly confident ❑ Very confident

Newer JAK Inhibitors

22. I believe the safety profile for the more selective JAKi targeting JAK1 (filgotinib and upadacitinib) is more favorable compared to tofacitinib (select one)

❑ Strongly disagree ❑ Disagree ❑ Undecided ❑ Agree ❑ Strongly agree

23. I will mitigate the risk factors for adverse events for the more selective JAKi targeting JAK1 (filgotinib and upadacitinib) in the same way as I currently do for tofacitinib (select one)

❑ Strongly disagree ❑ Disagree ❑ Undecided ❑ Agree ❑ Strongly agree

Thank you for taking your time participating in this survey
