# Supplementary material for: JAK inhibitors for the treatment of inflammatory bowel disease: results of an international survey of perceptions, attitudes, and clinical practice
Source: Eur J Gastroenterol Hepatol. 2023 Sep 17;35(11):1270–7. doi: 10.1097/MEG.0000000000002650 (PMC10659244; doi:10.1097/MEG.0000000000002650)
Supplement: Supplementary file 2 [file ejgh-35-1270-s002.pdf]

SUPPLEMENTARY TABLE 2: Univariate and multivariate analysis exploring prescribing factors for off-label JAKi use/where use cautioned

Prescribing JAKi in patients with existing VTE risk factors (except active IBD), if clinically appropriate

	Univariate analysis			Multivariate analysis	
	Refers for surgery before prescribing JAKi	Prescribes JAKi before referring for surgery	p-value	Odds Ratio (95% CI)	p-value
N	59	326			
Years trained, median (IQR)	10 (5-16)	9 (5-15)	0.72		
IBD cases/month, median (IQR)	65 (26-100)	60 (30-100)	0.83		
Practices in a tertiary centre, n (%)	37 (63)	240 (74)	0.11		
Practices in a university-affiliated hospital, n (%)	35 (59)	205 (63)	0.66		
Is an accredited gastroenterologist, n (%)	51 (86)	232 (71)	**0.02**	0.40 (0.17-0.85)	**0.02**
Prescribes JAKi at least monthly, n (%)	15 (25)	146 (45)	**0.01**	0.99 (0.99-1.02)	0.68
Is confident prescribing JAKi, n (%)	38 (64)	247 (76)	0.08		
Routinely places JAKi 3rd line/avoids unless only option, n (%)	46 (75)	174 (53)	**<0.01**	2.40 (1.25-4.9)	**0.01**
Would initiate alongside anticoagulation if appropriate, n (%)	2 (3)	92 (28)	**<0.01**	0.11 (0.02-0.36)	**<0.01**

Prescribing JAKi in patients already anticoagulated for another clinical reason, if clinically appropriate

	Univariate analysis			Multivariate analysis	
	Refers for surgery before prescribing JAKi	Prescribes JAKi before referring for surgery	p-value	Odds Ratio (95% CI)	p-value
N	79	306			
Years trained, median (IQR)	9 (5-16)	10 (5-15)	0.42		
IBD cases/month, median (IQR)	55 (26-100)	60 (30-100)	0.69		
Practices in a tertiary centre, n (%)	47 (59)	230 (75)	**0.01**	1.44 (0.76-2.74)	0.26
Practices in a university-affiliated hospital, n (%)	41 (52)	199 (65)	**0.04**	1.121 (0.60-2.07)	0.72
Is an accredited gastroenterologist, n (%)	61 (77)	222 (73)	0.48		
Prescribes JAKi at least monthly, n (%)	22 (28)	139 (45)	**<0.01**	1.42 (0.79-2.61)	0.25
Is confident prescribing JAKi, n (%)	47 (59)	238 (78)	**<0.01**	0.61 (0.35-1.10)	0.10
Routinely places JAKi 3rd line/avoids unless only option, n (%)	58 (73)	162 (53)	**<0.01**	0.57 (0.31-1.04)	0.07
Initiates JAKi in presence of VTE risk factors if appropriate, n (%)	9 (11)	37 (12)	>0.99		

Use of JAKi outside of marketing authorisation

	Univariate analysis			Multivariate analysis	
	Prescribes off label	Uses JAKi within product license only	p-value	Odds Ratio (95% CI)	p-value
N	121	264			
Practice is UK-based*, n (%)	34 (28)	55 (21)	0.12		
Practices in a tertiary centre, n (%)	105 (87)	172 (65)	**<0.01**	1.70 (0.85-3.46)	0.14
Practices in a university-affiliated hospital, n (%)	95 (79)	145 (55)	**<0.01**	1.68 (0.94-3.07)	0.09
Is an accredited gastroenterologist, n (%)	83 (69)	200 (76)	0.17		
Prescribes JAKi at least monthly, n (%)	74 (61)	87 (33)	**<0.01**	1.91 (1.15-3.18)	**0.01**
Is confident prescribing JAKi, n (%)	111 (92)	174 (66)	**<0.01**	4.04 (1.97-9.03)	**<0.01**
Routinely places JAKi 3rd line/avoids unless only option, n (%)	51 (42)	169 (64)	**<0.01**	1.34 (0.80-2.24)	0.27

*Crohn’s disease (CD) was included as an off-label use. At the time of survey distribution, the UK had just received marketing authorisation for upadacitinib for use in moderate to severe CD.
